# Assessing optimal methods for transferring machine learning models to low-volume and imbalanced clinical datasets: experiences from predicting outcomes of Danish trauma patients

**DOI:** 10.3389/fdgth.2023.1249258

**Published:** 2023-11-02

**Authors:** Andreas Skov Millarch, Alexander Bonde, Mikkel Bonde, Kiril Vadomovic Klein, Fredrik Folke, Søren Steemann Rudolph, Martin Sillesen

**Affiliations:** ^1^ Department of Organ Surgery and Transplantation, Copenhagen University Hospital, Rigshospitalet, Copenhagen, Denmark; ^2^ Center for Surgical Translational and Artificial Intelligence Research (CSTAR), Copenhagen University Hospital, Rigshospitalet, Copenhagen, Denmark; ^3^ Department of Computer Science, University of Copenhagen, Copenhagen, Denmark; ^4^ Copenhagen Emergency Medical Services, University of Copenhagen, Ballerup, Denmark; ^5^ Department of Cardiology, Herlev Gentofte University Hospital, Hellerup, Denmark; ^6^ Department of Anesthesia, Center of Head and Orthopedics, Rigshospitalet, Copenhagen, Denmark

**Keywords:** artificial intelligence, surgery, trauma, healthcare system, prediction model, transfer learning, mortality, length of stay

## Abstract

**Introduction:**

Accurately predicting patient outcomes is crucial for improving healthcare delivery, but large-scale risk prediction models are often developed and tested on specific datasets where clinical parameters and outcomes may not fully reflect local clinical settings. Where this is the case, whether to opt for de-novo training of prediction models on local datasets, direct porting of externally trained models, or a transfer learning approach is not well studied, and constitutes the focus of this study. Using the clinical challenge of predicting mortality and hospital length of stay on a Danish trauma dataset, we hypothesized that a transfer learning approach of models trained on large external datasets would provide optimal prediction results compared to de-novo training on sparse but local datasets or directly porting externally trained models.

**Methods:**

Using an external dataset of trauma patients from the US Trauma Quality Improvement Program (TQIP) and a local dataset aggregated from the Danish Trauma Database (DTD) enriched with Electronic Health Record data, we tested a range of model-level approaches focused on predicting trauma mortality and hospital length of stay on DTD data. Modeling approaches included de-novo training of models on DTD data, direct porting of models trained on TQIP data to the DTD, and a transfer learning approach by training a model on TQIP data with subsequent transfer and retraining on DTD data. Furthermore, data-level approaches, including mixed dataset training and methods countering imbalanced outcomes (e.g., low mortality rates), were also tested.

**Results:**

Using a neural network trained on a mixed dataset consisting of a subset of TQIP and DTD, with class weighting and transfer learning (retraining on DTD), we achieved excellent results in predicting mortality, with a ROC-AUC of 0.988 and an F2-score of 0.866. The best-performing models for predicting long-term hospitalization were trained only on local data, achieving an ROC-AUC of 0.890 and an F1-score of 0.897, although only marginally better than alternative approaches.

**Conclusion:**

Our results suggest that when assessing the optimal modeling approach, it is important to have domain knowledge of how incidence rates and workflows compare between hospital systems and datasets where models are trained. Including data from other health-care systems is particularly beneficial when outcomes are suffering from class imbalance and low incidence. Scenarios where outcomes are not directly comparable are best addressed through either de-novo local training or a transfer learning approach.

## Introduction

While clinical prediction models are used in a multitude of settings, determining the optimal approach for using these in healthcare systems where models were not trained is challenging (1). As such, in the situation where local datasets may be of a size potentially limiting the value of de-novo model training, whether to opt for this approach, direct porting of models trained on large non-local datasets or use other more recent approaches such as transfer learning, is not well explored.

An example of this clinical setting is traumatic injury due to violence and traffic accidents, where trauma volumes and outcomes differ substantially between different countries and healthcare systems (2). Trauma patients pose a dynamical, heterogenous population, often with complicated clinical trajectories where an abundance of biomedical data is generated in a short timeframe. While many prediction models have been fielded for providing clinicians with risk assessments of adverse outcomes in trauma patients (3–6) as well as other surgical cohorts (7), these are often developed on single-institution or national datasets with data only partly comparable to the local clinical setting where the model is used. As such, studies have indicated that such models often do not transfer well to other national or international hospital settings (8–11), presumably owing to the heterogeneous nature of the underlying patient population and treatment standard operating procedures (SOPs).

In contrast to legacy approaches, novel machine learning methods such as deep learning approaches have shown superior predictive performance in different surgical cohorts (12), but the choice of an optimal training strategy remains unsolved. This problem becomes especially pressing when models are deployed on datasets characterized by imbalanced data with low-probability outcomes (e.g., mortality in the trauma setting).

In a machine learning context, medical events such as trauma mortality are considered low probability outcomes with a prevalence of −4% of trauma cases (13), thus introducing class imbalance in the dataset. This can be addressed by using data-level methods to modify the training distribution and by model-level methods such as modifying class weighting in the learning process (14).

Using the clinical challenge of accurately predicting trauma mortality and hospital length-of-stay on a Danish trauma dataset characterized by class imbalance and limited data size, we investigated whether methods such as direct porting of an externally validated model trained on a large US trauma dataset, transfer learning or de-novo model training on local data would provide optimal risk prediction. In some cohorts, transfer learning has shown superiority to other strategies (15), and there are suggestions that site-specific customization is, in general, a key driver of predictive model performance (1, 16).

We hypothesized that a transfer learning approach would results in optimal performance. We furthermore hypothesized that using data-level approaches such as mixing training data between healthcare systems and implementing methods addressing class imbalance when relevant, could further improve performance.

## Methods

The study was approved by the regional data protection agency (approval #P-2020-180) the Danish Patient Safety Board (approval #31-1521-182), and the Danish Regions Quality Program (approval #DTDB-2021-09-23). Access to TQIP data was approved by the TQIP board of regents. In accordance with Danish law, informed consent is not required for retrospective observational studies without the need for interaction with patients, and this was thus not obtained.

The study was prepared in accordance with the Transparent Reporting of a multivariable prediction model for individual prognosis or diagnosis (TRIPOD) (17, 18).

### Data sources

In Denmark, healthcare is publicly funded and managed by five regions (19). All regions report clinical contact and outcome data to national clinical databases such as the Danish Trauma Registry (DTR). For this study we gained access to DTR and electronic health records (EHR) from two regions (Capital and Zealand regions) through the Danish Patient Authority for data registered from 2019 to 2021. Both regions employ the EPIC EHR system. DTR partially covers pre-hospital and in-hospital trajectories. The EHR data was used to enrich the dataset with in-hospital trajectory information (including outcomes) and provides comorbid conditions. The DTR and EHR was merged based on the Danish Central Person Registry (CPR) number, a unique identifier of every Danish citizen usable to identify individuals across EHR systems and clinical databases. The combined dataset will be referred to as the Danish Trauma Dataset (DTD).

For non-local data we acquired the 2017 version of the Trauma Quality Improvement Program (TQIP) by the American College of Surgeons (ACS). TQIP is available on request through the ACS website. This dataset will be referred to as the TQIP dataset (TQIPD).

### Inclusion

The Danish study population was identified by DTR inclusion criteria: Trauma patients who were received and initially treated through a trauma call (Danish procedure code BWST1F) in the National Patient Register (20) from 1st of January 2019 to 31st December 2021. Registrations missing datetime, registrations required for calculating length of stay, or missing either abbreviated injury severity score (AIS) or AIS derived injury severity score (ISS) were excluded.

The US population is defined by TQIP inclusion criteria (21).

### Outcomes

Primary outcome was all-cause in-hospital mortality.

Secondary outcome was long-term hospitalization, defined as a length of stay (LOS) longer than 2 days. This cut-off was chosen based on LOS distributions from both DTD and TQIPD, indicating that the majority of patients had shorter LOS. LOS was defined as the number of commenced calendar days from reception in the trauma care center till hospital discharge.

### Dataset construction

With tabular data, a machine learning model trained on a specific tabular dataset will only work on another dataset with the exact same features (22). In this study, we chose the largest possible intersection between the DTD and TQIPD based on availability and data quality.

Initially, we included variables from TQIPD available from the pre-hospital scene and within the first hour in-hospital. These included comorbid conditions, which can be obtained through health records registered prior to the trauma. After temporal filtering, we included variables that were obtainable in Danish data by similar data definitions as TQIPD. A complete list of included variables and value ranges is available in Supplementary Table S1.

### Overview of experimentation setup

For this study we chose to assess both model-level as well as data-level approaches.

In terms of model approaches, we assessed three different training strategies:

• De-novo training, meaning training and testing models on local (DTD) only.
• Porting, meaning training models on a non-local (TQIPD) dataset and directly exporting the models for testing on the local DTD test dataset without any altercations. For neural networks this means using unaltered weights and parameters from the TQIPD-based models.
• Transfer learning, meaning pre-training the neural network model on the non-local (TQIPD) dataset, subsequently fine-tuning the model with the Danish DTD dataset, and finally testing performance on the DTD test dataset.

Note, for comparability between approaches we used the exact same DTD test dataset with the structure and features from derived from TQIPD for all performance evaluation.

In terms of data-level approaches, we assessed three different strategies. These included:

• Training the models on a single training dataset (DTD or TQIPD where applicable)
• Training the models on a mixed training dataset (creating a training dataset including patients from both the DTD and TQIPD)
• Training models on a dataset created by implementing methods for countering class imbalance. When not using class weighting in the training process, we used resampling techniques. These included Synthetic Minority-Oversampling (SMOTE) and SMOTE in combination with under-sampling by removing Tomek links. Resampling was only performed on training data to avoid validating or testing on resampled data.

The first data-level scenario characterizes the setting where only local data is available, the second scenario where access to an external dataset is available, and the third scenario assesses the effects of implementing methods for countering class imbalance in a local dataset.

An overview of included models, dataset and resampling combinations for mortality models is available in Table 1. An overview of included models and datasets for long-term hospitalization is available in Table 2.

**Table 1:** Combinations of mortality prediction models and training datasets, which can be either Danish Trauma Dataset (DTD), the American Trauma Quality Improvement Program dataset (TQIPD) or a mixed traning dataset consisting of a random forest-selected subset of TQIPD and DTD training data (Mixed).

Model	Training data (retraining data)	Class imbalance methods
Trauma score and injury severity score (TRISS)	None	None
Random forest	Danish trauma dataset	Balanced mode
	Trauma quality improvement program dataset	SMOTE
	Mixed	SMOTETomek
AdaBoost	Danish trauma dataset	Balanced mode
	Trauma quality improvement program dataset	SMOTE
	Mixed	SMOTETomek
XGBoost	Danish trauma dataset	SMOTE
	Trauma quality improvement program dataset	SMOTETomek
	Mixed	
Explainable boosting machine	Danish trauma dataset	SMOTE
	Trauma quality improvement program dataset	SMOTETomek
	Mixed	
Neural network	Danish trauma dataset	Balanced weighting loss function
	Trauma quality improvement program dataset	SMOTE
	Mixed	SMOTETomek
Neural network (Transfer learning)	TQIPD/DTD	Balanced weighting loss function
	Mixed/DTD	SMOTE
		SMOTETomek

When a resampling method was applied on training data either Synthetic Minority Over-Sampling Technique (SMOTE) or SMOTE with down-sampling removing Tomek links was used (SMOTETomek).

**Table 2:** Combinations of long-term hospitalization models and training datasets, which can be either the Danish Trauma Dataset (DTD), the American Trauma Quality Improvement Program dataset (TQIPD) or a mixed training dataset consisting of a random forest-selected subset of TQIPD and DTD training data (Mixed).

Model	Training data (/retraining data)
Random forest	Danish trauma dataset
	Trauma quality improvement program dataset
	Mixed
AdaBoost	Danish trauma dataset
	Trauma quality improvement program dataset
	Mixed
XGBoost	Danish trauma dataset
	Trauma quality improvement program dataset
	Mixed
Explainable boosting machine	Danish trauma dataset
	Trauma quality improvement program dataset
	Mixed
Neural network	Danish trauma dataset
	Trauma quality improvement program dataset
	Mixed
Neural network (Transfer learning)	Trauma quality improvement program dataset/DTD
	Mixed/DTD

### Machine learning methods

We chose to include machine learning methods that do not heavily rely on assumptions of Gaussian distribution, allow adequate complexity and traditionally yield good performance on tabular data.

To establish a validated baseline for predicting mortality, we included the Trauma Score and Injury Severity Score (TRISS), which is commonly used for predicting mortality in trauma care (4). As a machine learning baseline model, we trained a random forest classifier [using the Scitkit-learn implementation (23)] per outcome being the simplest model selected. Random forest models served as base estimator for AdaBoost (24) classifiers. We also selected XGBoost (25) for testing a gradient boosting framework, which is less sensitive to noisy data and outliers than Adaboost and typically also less prone to overfitting (26). We included InterpretMLs Explainable Boosting Machine (EBM) (27) for the frameworks built-in focus on explainable model decision making. EBM is a tree-based, cyclic gradient boosting Generalized Additive Model which comparatively is slow due to round-robin fashion cycling through features but delivers performance on parr with “black box” state of the art methods (27). For neural networks we chose the FastAI library (28) which provides a high-level framework of the PyTorch package (29) with built-in support for tabular data.

### Model configurations and hyper-parameters

Below the general model configuration of hyper-parameters used across models are briefly described. Complete configurations for all models are available in the referenced GitHub repository.

#### Random forest

The number of estimators was 100 using the Gini impurity criterion for measuring the quality of a split. Max depth of the trees was not set, requiring a minimum of 2 samples to split an internal node and 5 samples to be a leaf node. When looking for the best split, the model considered the square root of number of features. To address class imbalance with mortality as outcome we used balanced class weighting by adjusting weights inversely proportional to class frequencies in the training data.

#### AdaBoost

Using the random forest model as base model AdaBoost (24) inherited hyper-parameters using SAMME.R boosting algorithm (30). The learning rate was at maximum 0.4.

#### XGBoost

The XGBoost models (25) used the GBTree boosting algorithm with learning rate at 0.3 with a maximum depth of the tree of 10 using default regularization. The evaluation metric was area under the receiver operator characteristics curve for long-term hospitalization and area under precision recall curve for mortality.

#### Explainable boosting model (EBM)

The EBM classifier (27) was allowed a maximum of 64 bins per feature for built-in pre-processing stage to reduce overfitting. A maximum of 5 leaf nodes was used in a maximum of 5,000 boosting rounds with early stopping after 50 rounds without improvement. Both inner and outer bags were set to 25 for marginally higher accuracy at costs of slower training time. When training models on full TQIPD inner bags was 8 and outer bags 0 (default values) for reduced training time.

#### Neural network architecture

Categorical input variables were stacked in embedding matrixes with a 4% dropout probability layer. Both categorical and continuous input variables were passed through a batch normalization layer to the first linear layer with 200 nodes and a rectified linear unit layer with initial dropout probability of 1%. Each following linear layer uses batch normalization and a dropout probability of 0.1% with reductive size from 100 to 20. The final layer had no batch normalization ending with two nodes for either positive or negative outcome.

The model used the Adam optimizer with a flattened binary cross entropy loss function with class weighting calculated using the inverse proportion of class frequencies in training data. Weight decay was set to 0.2. When training on mixed or TQIPD dataset batch size was 1,024 and training (or retraining) on DTD batch size was 64. The learning rate was selected from the FastAI package (28) built-in learning rate finder by using the mid-point between slide and valley multiplied by a factor of 0.5 when retraining during transfer learning. Models were allowed to train for up to 5 epochs with early stopping callback based on either recall score or validation loss depending on the model.

### Pre-processing

For both TQIPD and DTD we removed outliers (faulty registrations) in continuous outputs by feature specific boundaries (Supplementary Table S1). For height and weight unit conversion was attempted before removing if still out of bounds. Categorical features were technically categorified after any non-valid categories were replaced with nulls. The primary external cause of injury is ICD-10 coded in TQIPD. These were mapped to match DTD where following categories are used: Accident, assault, self-harm or other. In both datasets AIS-codes were one-hot encoded by severity of each region.

For neural network models, missing values were replaced by mode with an additive binary variable indicating missing value. Continuous data were normalized by mean and standard deviation. For categorical data we used entity embeddings stacked into embedding matrices.

For non-neural network models missing values were replaced with mode for both categorical and continuous inputs. No pretraining normalization was done for continuous inputs.

**Figure 1:**
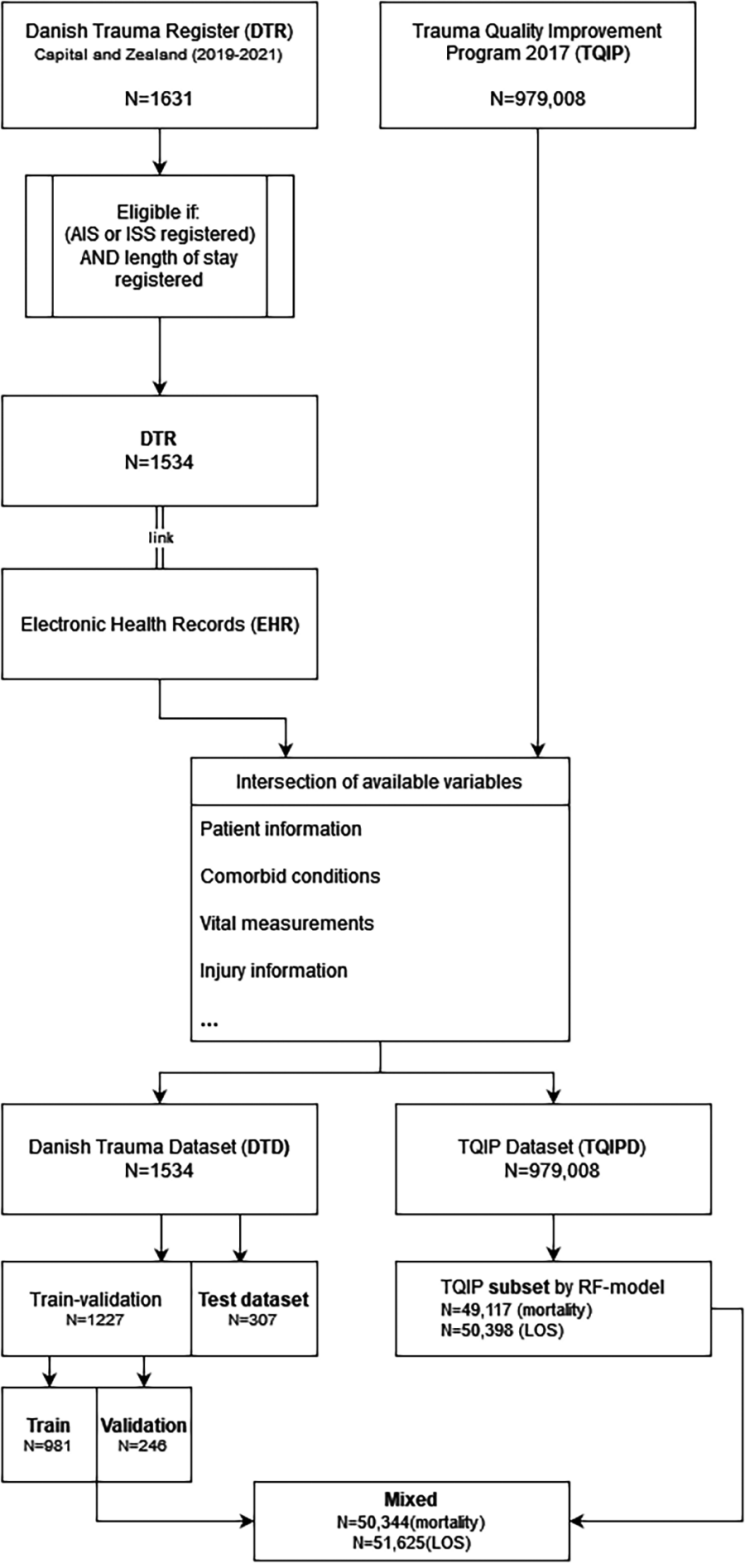
Population and dataset construction flow-chart.

We reserved 20 percent of DTD as test dataset after pre-processing for all model performance evaluations (Figure 1). The remainder of DTD was split into 80% training data and 20% validation data when models were exclusively trained on DTD. The proportion between training and validation datasets was 80% training and 20% validation throughout all models.

Two subsets of TQIPD were selected for mixing with the DTD training and validation subset Intending to select data from TQIPD with the highest similarity to DTD, we trained a random forest model per outcome to predict if a row originated from DTD or TQIPD. The data-selection model was trained with a randomly selected subset of TQIPD mixed with the entire DTD. The model was used to predict for every case in the entire TQIPD, if the case was Danish or not. TQIPD cases that were predicted to be Danish were selected and mixed with DTD.

### Metrics for performance evaluation

While receiver operating characteristic (ROC) curve and ROC area under curve (AUC) are commonly applied as evaluation metrics to summarize the performance of a binary classification model, we chose to include precision and recall (PR) due to class imbalance with mortality as outcome. In ROC-space the relation between true positive rates and false positive rates is apparent but with no representation of false negatives. The false positive rate will be unfairly represented where true negatives considerably outnumber true positives. In PR-space we compare false positives to true positives and false negatives to true negatives (31). This provides important insights into model behavior in problems with rare occurrence of one class such as mortality in trauma.

For the clinical purpose of a mortality prediction model specifically, we wanted to prioritize sensitivity (recall) over precision. The consequence of inaction, in this case, is larger than the cost of overreacting when the purpose for a clinician interpreting individualized mortality risk is to decide on resource allocation, choosing adequately advanced treatment facilities and administering prophylactic treatments. Precision and recall were summarized as F-2 score for mortality due to class imbalance and as F-1 score for long-term hospitalization.

F-1 score is the harmonic mean of precision and recall (32):

$$F1=2\frac{\mathrm{precision}\;\;\times \;\mathrm{recall}}{\mathrm{precision}\;+\;\mathrm{recall}}$$

Where F-2 score is *Fβ* using factor 2, where the *β*. factor considers recall *β* times as important as precision (32):

$$F\beta =(1+\beta^{2})\frac{\mathrm{precision}\;\;\times \;\mathrm{recall}}{(\beta^{2}\;\times \;\mathrm{precision})+\mathrm{recall}}$$

The default probability threshold for assigning classes is 0.5; however generally suboptimal for imbalanced datasets. We therefore used *F*-beta optimization on validation data for threshold-moving on test data probabilities.

### Model behavior

For tree-based models, we used the default Scik-learn feature importance module, which is based on mean decrease in impurity. For neural networks, we used Shapley Additive Explanations (SHAP) (33). The average of the marginal contributions across all permutations (SHAP values) was calculated for the DTD test population presenting the 20 features with the highest impact on model output magnitude.

For EBM models, we used the built-in features to display the 15 features with the highest overall importance based on predictions for DTD test population.

### Data presentations

Data are presented as medians [interquartile range] or percentages, where appropriate.

### Implementations

All models were implemented using Python version 3.8.5. Scikit-learn (23) version 1.1.2 were used for Random Forest models, AdaBoost, calculating metrics and feature importance. XGBoost (25) was version 1.3.3 and InterpretML (27) version 0.2.7 for Explainable Boosting Classifier. For neural networks we used FastAI (28) version 2.7.9 based on PyTorch (29) version 1.10.2. Imbalanced-learn (34) version 0.7.0 was used for resampling techniques and SHAP (33) 0.41.0 for Shapley values.

## Results

The complete Danish patient study population DTD consisted of 1,534 cases with 121 deceased patients and 1,081 long-term hospitalized patients (Figure 1). Demographic information is presented in Table 3. The median age was 38.5 years with 72.4% males. The primary cause of injury was in 68% of DTD cases accidental (such as vehicular accidents), 10.9% assault, 3.5% self-harm and 17.7% other (poisoning, drowning etc.). The median Injury Severity Score (ISS) was 10 [4–20]. Detailed characteristics and incidences are available in Table 3.

**Table 3:** Overview of demographic and difference in variables between outcome groups for danish trauma dataset and trauma quality improvement program dataset.

		Danish trauma dataset		Trauma quality improvement program dataset	
		Missing	Overall	Missing	Overall
*n*			1,534		979,008
Sex (female, male), *n* (%)	Female	0	424 (27.6)	140	
	Male		1,110 (72.4)		
Hospital teaching status, *n* (%)	community	0	58 (3.8)	4,158	4,158
	university		1,476 (96.2)		4,40,012 (45.1)
	non-teaching		0 (0.0)		162,893 (16.7)
Hospital count of beds, *n* (%)	≤200	0		0	91,854 (9.4)
	201−400		11 (0.7)		320,438 (32.7)
	401–600		45 (2.9)		260,601 (26.6)
	>600		1,478 (96.3)		306,115 (31.3)
Hospital ACS trauma center Verification level, *n* (%)	I - Level I trauma center	0	1,465 (95.5)	302,846	396,968 (58.7)
	II - Level II trauma center		69 (4.5)		217,169 (32.1)
	III - Level III trauma center		0 (0.0)		62,025 (9.2)
Comorbid condition: ADHD, *n* (%)	0	0	1,514 (98.7)	0	966,884 (98.8)
	1		20 (1.3)		12,124 (1.2)
Comorbid condition: Alcoholism, *n* (%)	0	0	1,452 (94.7)	0	927,637 (94.8)
	1		82 (5.3)		51,371 (5.2)
Comorbid condition: Angina pectoris, *n* (%)	0	0	1,528 (99.6)	0	977,966 (99.9)
	1		6 (0.4)		1,042 (0.1)
Comorbid condition: Anticoagulant treatment, *n* (%)	0	0	1,531 (99.8)	0	906,648 (92.6)
	1		3 (0.2)		72,360 (7.4)
Comorbid condition: Bleeding disorders, *n* (*n* (%)	0	0	1,533 (99.9)	0	963,933 (98.5)
	1		1 (0.1)		15,075 (1.5)
Comorbid condition: Chemotherapy, *n* (%)	0	0	1,525 (99.4)	0	975,569 (99.6)
	1		9 (0.6)		3,439 (0.4)
Comorbid condition: Cirrhosis, *n* (%)	0	0	1,529 (99.7)	0	969,537 (99.0)
	1		5 (0.3)		9,471 (1.0)
Comorbid condition: Chronic obstructive pulmonary disease, *n* (%)	0	0	1,517 (98.9)	0	918,739 (93.8)
	1		17 (1.1)		60,269 (6.2)
Comorbid condition: Cerebrovascular accident, *n* (%)	0	0	1,484 (96.7)	0	954,219 (97.5)
	1		50 (3.3)		24,789 (2.5)
Comorbid condition: Dementia, *n* (%)	0	0	1,528 (99.6)	0	928,010 (94.8)
	1		6 (0.4)		50,998 (5.2)
Comorbid condition: Diabetes (type 1 and 2), *n* (%)	0	0	1,494 (97.4)	0	858,863 (87.7)
	1		40 (2.6)		120,145 (12.3)
Comorbid condition: Congestive heart failure, *n* (%)	0	0	1,525 (99.4)	0	942,578 (96.3)
	1		9 (0.6)		36,430 (3.7)
Comorbid condition: Hypertension, *n* (%)	0	0	1,460 (95.2)	0	673,942 (68.8)
	1		74 (4.8)		305,066 (31.2)
Comorbid condition: Myocardial infarction, *n* (%)	0	0	1,531 (99.8)	0	970,481 (99.1)
	1		3 (0.2)		8,527 (0.9)
Comorbid condition: Peripheral arterial disease, *n* (%)	0	0	1,530 (99.7)	0	974,097 (99.5)
	1		4 (0.3)		4,911 (0.5)
Comorbid condition: Mental disorders (schizophrenia, bipolar disorder, major depressive disorder, social anxiety, PTSD, antisocial personality disorder), *n* (%)	0	0	1,457 (95.0)	0	886,995 (90.6)
	1		77 (5.0)		92,013 (9.4)
Comorbid condition: Chronic renal failure, *n* (%)	0	0	1,532 (99.9)	0	964,037 (98.5)
	1		2 (0.1)		14,971 (1.5)
Comorbid condition: Smoking tobacco every day or some days within past 12 months, *n* (%)	0	0	1,529 (99.7)	0	804,311 (82.2)
	1		5 (0.3)		174,697 (17.8)
Comorbid condition: Documented substance abuse (cannabis, hallucinogens, inhalents, opiods, sedatives, other), *n* (%)	0	0	1,532 (99.9)	0	928,955 (94.9)
	1		2 (0.1)		50,053 (5.1)
Primary cause of injury, *n* (%)	accident	0	1,043 (68.0)	0	834,733 (85.3)
	assault		167 (10.9)		91,661 (9.4)
	other		271 (17.7)		39,308 (4.0)
	self_harm		53 (3.5)		13,306 (1.4)
Age in years, median [Q1,Q3]		0	38.5 [22.0,58.0]	59,884	49.0 [26.0,69.0]
Weight in kg, median [Q1,Q3]		316	75.6 [63.0,87.9]	500,242	75.0 [61.0,90.0]
Height in cm, median [Q1,Q3]		477	175.0 [168.0,182.0]	521,164	170.0 [160.0,178.0]
Pre-hospital systolic blood pressure, median [Q1,Q3]		357	125.0 [104.0,144.0]	445,831	138.0 [120.0,156.0]
Pre-hospital Glasgow coma score (total), median [Q1,Q3]		214	15.0 [12.0,15.0]	442,619	15.0 [15.0,15.0]
In-hospital Glasgow coma score (total), median [Q1,Q3]		65	15.0 [14.0,15.0]	51,607	15.0 [15.0,15.0]
In-hospital systolic blood pressure, median [Q1,Q3]		15	129.0 [110.0,142.5]	33,156	136.0 [120.0,154.0]
In-hospital pulserate (BPM), median [Q1,Q3]		18	88.0 [77.0,102.0]	22,293	87.0 [74.0,101.0]
In-hospital temperature in celsius, median [Q1,Q3]		337	36.9 [36.4,37.2]	99,890	36.7 [36.4,36.9]
In-hospital pulseoximetry, median [Q1,Q3]		15	99.0 [97.0,100.0]	43,408	98.0 [96.0,100.0]
In-hospital respiratory rate (BPM), median [Q1,Q3]		105	16.0 [14.0,20.0]	34,246	18.0 [16.0,20.0]
Injury severity score derived from AIS, median [Q1,Q3]		88	10.0 [4.0,20.0]	3,204	8.0 [4.0,10.0]
Absolute difference between pre- and in-hospital Glasgow coma score, median [Q1,Q3]		214	0.0 [0.0,0.0]	462,814	0.0 [0.0,0.0]
Absolute difference between pre- and in-hospital systolic blood pressure measurement, median [Q1,Q3]		366	−4.0 [−20.0,10.0]	455,628	0.0 [−15.0,14.0]
Long-term hospitalization (%)		0	1,081 (0.7)	0	623,345 (0.64)
In-hospital mortality (%)		0	121 (0.08)	0	32,400 (0.03)

In total, 1,227 DTD cases were used for training and validation and 307 cases reserved for test data. In training and validation data 99 patients were deceased and 866 patients long-term hospitalized, leaving 22 deceased in test data and 215 long-term hospitalizations. An overview of LOS distribution in commenced days for DTD is available in Supplementary Figure S1A.

The total TQIPD population consisted of 979,008 cases with 32,400 deceased patients and 623,345 long-term hospitalizations. An overview of LOS distribution in commenced days for TQIPD is available in Supplementary Figure S1B. A total of 50,000 TQIPD cases were selected randomly for a secondary mixed dataset. A total of 49,117 cases for mortality models and another 50,398 cases for long-term hospitalization models were selected from TQIPD using a random forest model and mixed with the DTD train-validation dataset (referred to as “mixed dataset” in the following).

### Performance—mortality models

**Figure 2:**
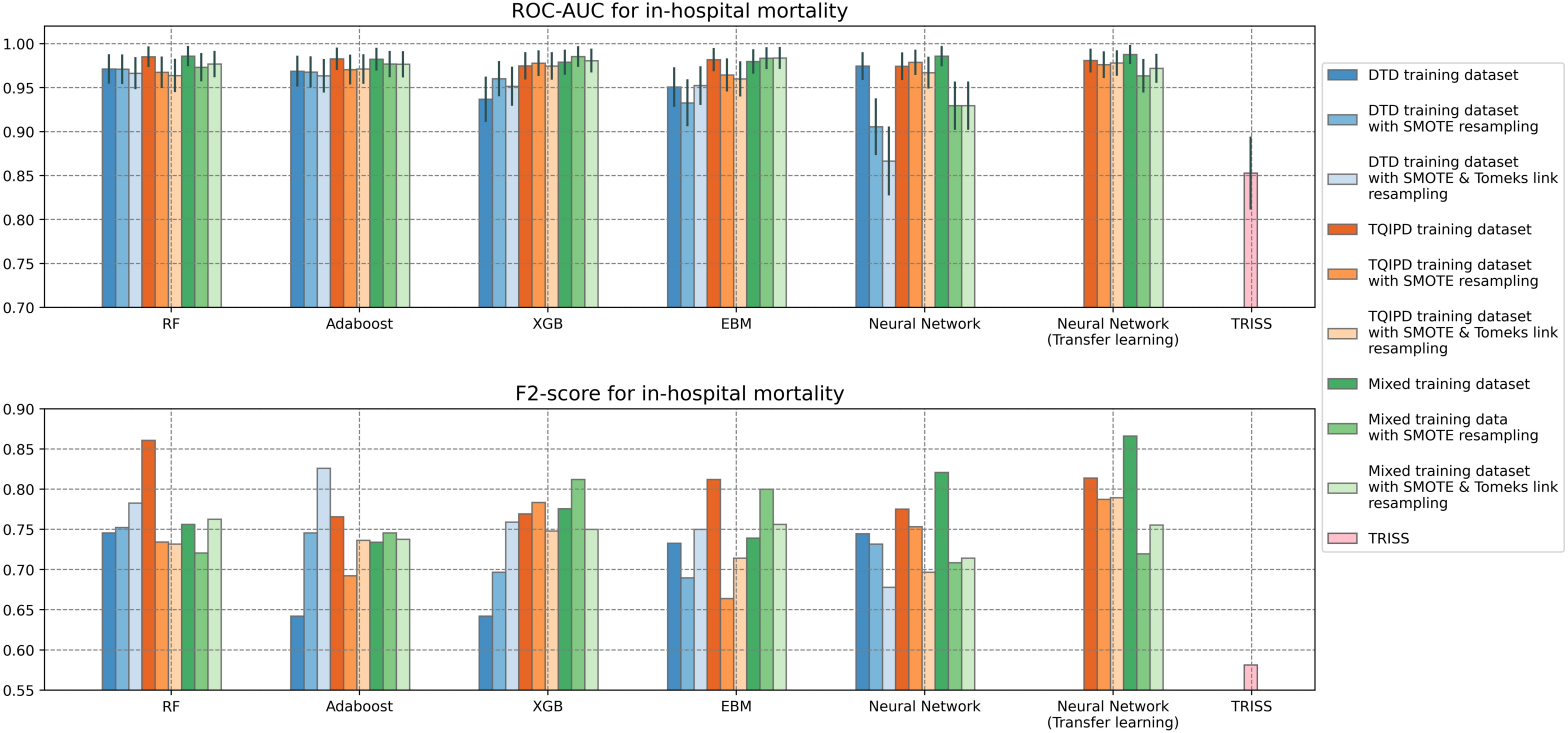
Area under the receiver operator characteristics curve (ROC-AUC) with confidence interval (grey vertical line) and F2-scores for all mortality models using trauma score and injury severity score (TRISS) as baseline. Comparing Random Forrest model (RF), AdaBoost, XGBoost (XGB), Explainable Boosting Machine (EBM) and neural networks trained on either Danish Trauma Dataset (DTD), the American Trauma Quality Improvement Program dataset (TQIPD), a mixed traning dataset consisting of a RF-selected subset of TQIPD and DTD training data (Mixed) or a randomly selected subset from TQIPD mixed with DTD training data (Mixed randomly). When a resampling method was applied on training data either Synthetic Minority Over-Sampling Technique (SMOTE) or SMOTE with down-sampling removing Tomek links was used (SMOTETomek). When class weighting was applied on a neural network loss function, weights are denoted first by the negative class and secondly for the postive class.

**Figure 3:**
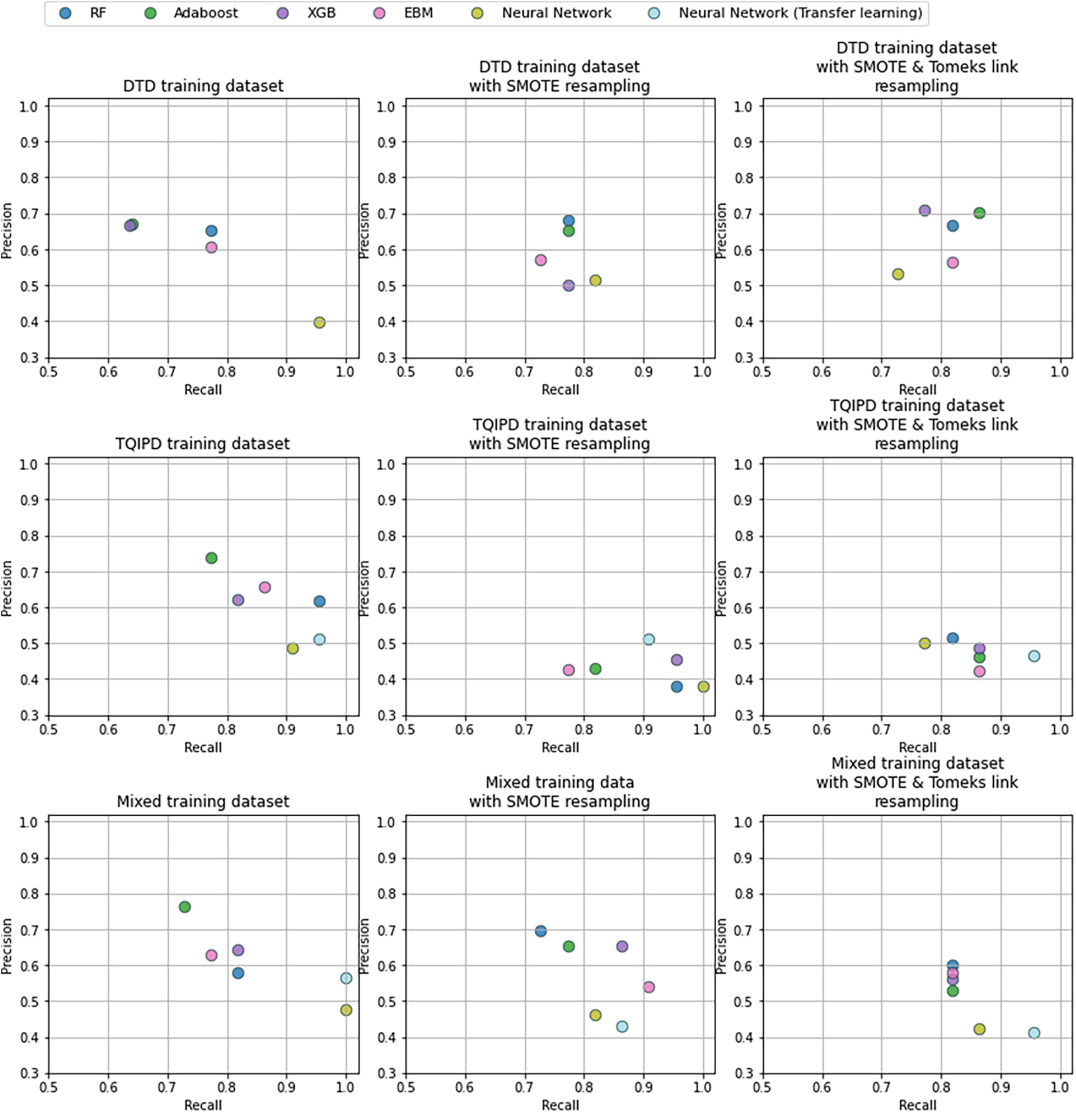
Precision and recall -scores for mortality models comparing random forrest model (RF), adaBoost, XGBoost (XGB), explainable boosting machine (EBM) and neural networks trained on either danish trauma dataset (DTD), the American trauma quality improvement program dataset (TQIPD) or a mixed traning dataset consisting of a RF-selected subset of TQIPD and DTD training data (mixed). When a resampling method was applied on training data either Synthetic Minority Over-Sampling Technique (SMOTE) or SMOTE with down-sampling removing Tomek links was used (SMOTETomek). When class weighting was applied on a neural network loss function, weights are denoted first by the negative class and secondly for the postive class.

Performance metrics for all mortality models are presented as ROC-AUC and F2-scores in Figure 2 and as precision-recall in Figure 3. A table of all performance metrics for mortality models are presented in Table 4.

**Table 4:** Performance metrics, including confidence intervals for area under the receiver operater characteristics curve (ROC-AUC), for all mortality models using trauma score and injury severity score (TRISS) as baseline, comparing models trained on either danish trauma dataset (DTD), the American trauma quality Improvement program dataset (TQIPD) or a mixed traning dataset consisting of a random forest-selected subset of TQIPD and DTD training data (mixed). Neural network with transfer learning was retrained on DTD. When a resampling method was applied on training data either Synthetic Minority Over-Sampling Technique (SMOTE) or SMOTE with down-sampling removing Tomek links was used (SMOTETomek). When class weighting was applied on a neural network loss function, weights are denoted first by the negative class and secondly for the postive class.

Model	Training data source	Resampling method	Weights	ROC-AUC	Lower CI ROC-AUC	Upper CI ROC-AUC	F2 score	Precision	Recall
TRISS	DTD	None	None	0.853	0.751	0.955	0.581	0.251	0.867
RF	DTD	None	None	0.971	0.922	1.0	0.746	0.654	0.773
		SMOTE	None	0.971	0.922	1.0	0.752	0.680	0.773
		SMOTETomek	None	0.966	0.913	1.0	0.783	0.667	0.818
	Mixed	None	None	0.986	0.951	1.0	0.756	0.581	0.818
		SMOTE	None	0.973	0.926	1.0	0.721	0.696	0.727
		SMOTETomek	None	0.977	0.933	1.0	0.763	0.600	0.818
	TQIPD	None	None	0.985	0.949	1.0	0.861	0.618	0.955
		SMOTE	None	0.967	0.915	1.0	0.734	0.382	0.955
		SMOTETomek	None	0.964	0.909	1.0	0.732	0.514	0.818
AdaBoost	DTD	None	None	0.969	0.918	1.0	0.642	0.667	0.636
		SMOTE	None	0.968	0.916	1.0	0.746	0.654	0.773
		SMOTETomek	None	0.963	0.908	1.0	0.826	0.704	0.864
	Mixed	None	None	0.982	0.943	1.0	0.734	0.762	0.727
		SMOTE	None	0.977	0.933	1.0	0.746	0.654	0.773
		SMOTETomek	None	0.977	0.932	1.0	0.738	0.529	0.818
	TQIPD	None	None	0.983	0.944	1.0	0.766	0.739	0.773
		SMOTE	None	0.970	0.921	1.0	0.692	0.429	0.818
		SMOTETomek	None	0.971	0.922	1.0	0.736	0.463	0.864
XGB	DTD	None	None	0.937	0.866	1.0	0.642	0.667	0.636
		SMOTE	None	0.960	0.903	1.0	0.697	0.500	0.773
		SMOTETomek	None	0.952	0.889	1.0	0.759	0.708	0.773
	Mixed	None	None	0.979	0.937	1.0	0.776	0.643	0.818
		SMOTE	None	0.985	0.950	1.0	0.812	0.655	0.864
		SMOTETomek	None	0.981	0.940	1.0	0.750	0.562	0.818
	TQIPD	None	None	0.975	0.929	1.0	0.769	0.621	0.818
		SMOTE	None	0.978	0.934	1.0	0.784	0.457	0.955
		SMOTETomek	None	0.975	0.928	1.0	0.748	0.487	0.864
EBM	DTD	None	None	0.951	0.887	1.0	0.733	0.607	0.773
		SMOTE	None	0.933	0.859	1.0	0.690	0.571	0.727
		SMOTETomek	None	0.952	0.890	1.0	0.750	0.562	0.818
	Mixed	None	None	0.980	0.938	1.0	0.739	0.630	0.773
		SMOTE	None	0.984	0.946	1.0	0.800	0.541	0.909
		SMOTETomek	None	0.984	0.946	1.0	0.756	0.581	0.818
	TQIPD	None	None	0.982	0.942	1.0	0.812	0.655	0.864
		SMOTE	None	0.964	0.910	1.0	0.664	0.425	0.773
		SMOTETomek	None	0.960	0.902	1.0	0.714	0.422	0.864
Neural network	DTD	None	0.5439, 6.1970	0.974	0.928	1.0	0.745	0.396	0.955
		SMOTE	None	0.906	0.821	0.991	0.732	0.514	0.818
		SMOTETomek	None	0.867	0.769	0.964	0.678	0.533	0.727
	Mixed	None	0.5503, 5.4705	0.986	0.951	1.0	0.821	0.478	1.0
		SMOTE	None	0.930	0.855	1.0	0.709	0.462	0.818
		SMOTETomek	None	0.930	0.855	1.0	0.714	0.422	0.864
	TQIPD	None	None	0.974	0.928	1.0	0.775	0.488	0.909
		SMOTE	None	0.979	0.936	1.0	0.753	0.379	1.0
		SMOTETomek	None	0.967	0.914	1.0	0.697	0.500	0.773
Neural network (Transfer learning)	Mixed	None	0.5503, 5.4705	0.988	0.955	1.0	0.866	0.564	1.000
		SMOTE	None	0.963	0.908	1.0	0.720	0.432	0.864
		SMOTETomek	None	0.972	0.923	1.0	0.755	0.412	0.955
	TQIPD	None	None	0.981	0.940	1.0	0.814	0.512	0.955
		SMOTE	None	0.976	0.931	1.0	0.787	0.513	0.909
		SMOTETomek	None	0.978	0.935	1.0	0.789	0.467	0.955

The highest ROC-AUC was 0.988 by using class weighting and transfer learning in a neural network pretrained with the mixed dataset. However, several other models achieved −0.98 ROC-AUC as well (Table 4). Based on ROC-AUC all models performed well and superior to TRISS (ROC-AUC: 0.853).

The highest F2-score was of 0.866 also using class weighting and transfer learning in a neural network pretrained with the mixed dataset. Porting a TQIPD-based random forest model achieved 0.861 F2-score. Using a DTD-based de-novo AdaBoost model applying Synthetic Minority-Oversampling Technique (SMOTE) and under-sampling by removing Tomek links the F2-score was 0.826. In comparison, TRISS had a F2-score of 0.581.

Inspecting these three models by precision and recall, the transfer learning neural network model achieved perfect recall and 0.564 precision. The ported random forest model also scored near perfect recall (0.955) with high precision (0.618). The de-novo AdaBoost scored 0.864 recall and favored precision with a score of 0.704. Comparably TRISS scored 0.867 recall and 0.251 precision.

### Model behavior—mortality

For the well-performing transfer learning model, we calculated mean SHAP values for predictions on DTD test data as in Supplementary Figure S2. Age, ISS and GCS were of highest global impact.

We extracted feature importance for the de-novo AdaBoost model displayed in Supplementary Figure S3. Glasgow Coma Score (both from EMS and in-hospital), injury severity score (ISS), systolic blood pressure (SBP) and age was of most importance. Similar for the ported random forest model the highest-ranking features were GCS, ISS, in-hospital SBP, critical head injury, in-hospital pulse rate and age (Supplementary Figure S4).

For comparison, we included the overall importance summary of mean absolute scores from the Explainable Boosting Machine model trained on mixed SMOTE and Tomek links resampled data (Supplementary Figure S5). This showed the overlap in features ranked as most important with GCS, ISS, age, SBP but also pulse oximetry and pulse rate.

### Performance—long-term hospitalization models

**Figure 4:**
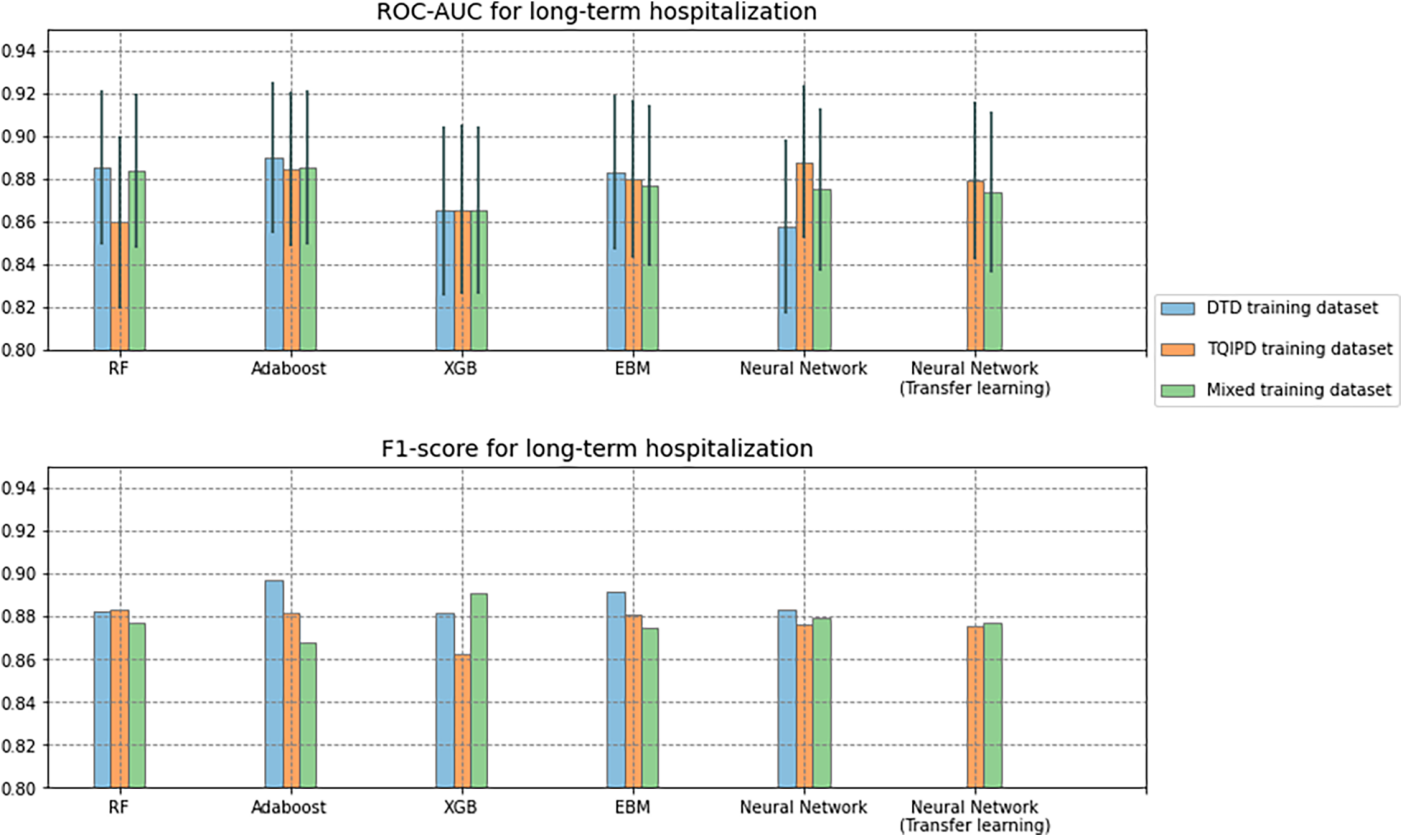
Area under the receiver operator characteristics curve (ROC-AUC) with confidence interval (grey vertical line) and F1-scores for all long-term hospitalization models. Comparing Random Forrest model (RF), AdaBoost, XGBoost (XGB), Explainable Boosting Machine (EBM) and neural networks trained on either Danish Trauma Dataset (DTD), the American Trauma Quality Improvement Program dataset (TQIPD) or a mixed traning dataset consisting of a RF-selected subset of TQIPD and DTD training data (Mixed). Neural networks with transfer learning are always retrained on DTD.

**Figure 5:**
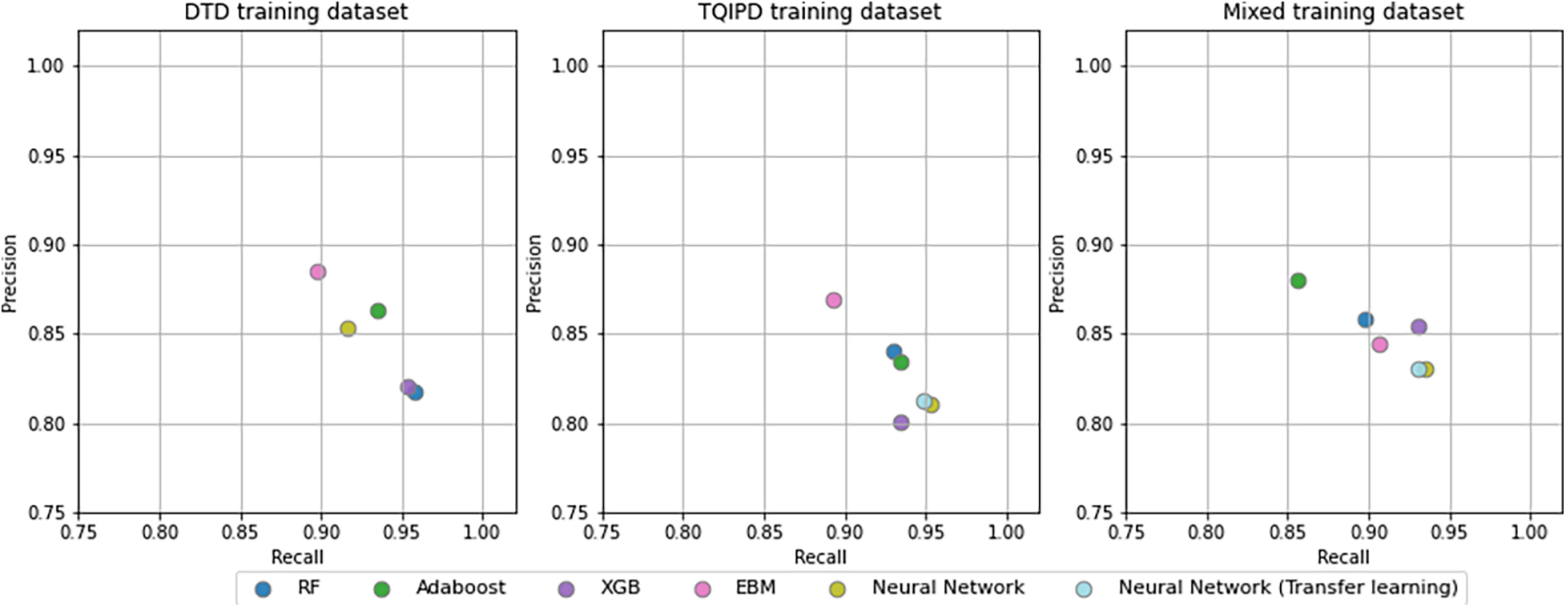
Precision and recall -scores for all mortality models comparing random forrest model (RF), adaBoost, XGBoost (XGB), explainable boosting machine (EBM) and neural networks trained trained on either danish trauma dataset (DTD), the American trauma quality improvement program dataset (TQIPD) or a mixed traning dataset consisting of a RF-selected subset of TQIPD and DTD training data (mixed). Neural networks with transfer learning are always retrained on DTD.

Performance metrics for all long-term hospitalization models are presented as ROC-AUC and F1-scores in Figure 4 and as precision-recall in Figure 5. A table of all metrics is presented in Table 5.

**Table 5:** Performance metrics, including confidence intervals for area under the receiver operater characteristics curve (ROC-AUC), for all long-term hospitalization models comparing models trained on either danish trauma dataset (DTD), the American trauma quality Improvement program dataset (TQIPD) or a mixed traning dataset consisting of a random forest-selected subset of TQIPD and DTD training data (mixed). Neural network with transfer learning was retrained on DTD.

Model	Training data set	ROC-AUC	Lower CI ROC-AUC	Upper CI ROC-AUC	Precision	Recall	F1 score
Random forest	DTD	0.885	0.849	0.921	0.817	0.958	0.882
	TQIPD	0.860	0.820	0.900	0.840	0.930	0.883
	Mixed	0.884	0.848	0.920	0.858	0.898	0.877
AdaBoost	DTD	0.890	0.855	0.925	0.863	0.935	0.897
	TQIPD	0.885	0.849	0.921	0.834	0.935	0.822
	Mixed	0.884	0.848	0.920	0.858	0.898	0.877
XGBoost	DTD	0.865	0.825	0.904	0.820	0.953	0.882
	TQIPD	0.866	0.826	0.905	0.801	0.935	0.863
	Mixed	0.865	0.826	0.905	0.855	0.930	0.891
Explainable boosting machine	DTD	0.883	0.847	0.919	0.885	0.898	0.891
	TQIPD	0.880	0.843	0.917	0.869	0.893	0.881
	Mixed	0.877	0.839	0.914	0.844	0.907	0.874
Neural network	DTD	0.857	0.817	0.898	0.853	0.916	0.883
	TQIPD	0.888	0.852	0.923	0.810	0.953	0.876
	Mixed	0.875	0.837	0.913	0.831	0.935	0.88
Neural network (Transfer learning)	TQIPD/DTD	0.879	0.842	0.916	0.813	0.949	0.876
	Mixed/DTD	0.874	0.836	0.912	0.830	0.930	0.877

All models had at least one configuration resulting in at least 0.870 ROC-AUC. AdaBoost, XGBoost, Neural network (transfer learning) and EBM all scored −0.89 F1-score. All models scored higher than 0.800 precision and higher than 0.890 recall. The DTD-based de-novo EBM model reached the highest precision of 0.885 and the highest recall was the de-novo DTD-based random forest model with 0.958.

### Model behavior—long-term hospitalization models

We extracted feature importance from the de-novo random forest model as displayed in Supplementary Figure S6. ISS was dominantly important followed by age, weight, in-hospital temperature measurement, head injury (derived from AIS), thoracic injury, and in-hospital SBP measurement.

For the mixed dataset-based transfer learning neural network, the highest SHAP values were also dominantly ISS, followed by moderate and serious injury to lower extremities, age, moderate spine and upper extremity injury, and GCS (Supplementary Figure S7).

Lastly, the DTD-based de-novo EBM model had the highest mean absolute score for ISS, followed by in-hospital temperature measurement, weight, in-hospital respiratory rate, moderate thorax injury, and age (Supplementary Figure S8).

## Discussion

In this study, we aimed to identify optimal machine learning approaches for predicting clinical outcomes on local low-volume and imbalanced tabular datasets, using the clinical scenario of predicting outcomes for Danish trauma patients as a practical use case. Predicting both mortality and long-term hospitalization was achievable with good performance on DTD test dataset by training models on both a limited local dataset, a dataset from another healthcare system and by mixing a dataset of both local and non-local data.

We hypothesized that a transfer learning approach would result in optimal performance while addressing class imbalance. When predicting mortality, including non-local (TQIPD) data in the training process, regardless of methods e.g., mixing training sets or subsequent fine-tuning, showed better performance compared to similar model architectures trained only on local DTD data. Addressing class imbalance on algorithm level (class weighting) showed similar or marginally better performance, while being computationally more efficient that data resampling. Assessing long-term hospitalization prediction models our results indicated no benefit from including non-local data with very similar performance between model architectures.

Assessing mortality prediction models primarily by sensitivity and specificity, the best-performing model seemed to vary between all three model-level approaches. For de-novo training on DTD data, optimal performance was achieved using an AdaBoost model with data resampling. For porting, a random forest model trained on the TQIPD without resampling was best performing. For mixed dataset-based models, a neural network using transfer learning with class weighting resulted in the best performance, and the best performance between all models in general.

However, it is important to note that ROC-AUC confidence intervals were overlapping between our models indicating no statistically significant difference.

Danish Trauma patients represent a heterogeneous patient cohort from a well-developed but small healthcare system. Due to these constraints, Danish trauma patient datasets will naturally be of limited size but characterized by patients suffering from infrequent yet high-impact complications (e.g., mortality rates). In a real-world clinical scope, our results suggest that when prediction models are fielded in a setting such as Danish Trauma care, targeting outcomes that have comparable incidence rates between healthcare systems (e.g., trauma mortality, which is comparable between the TQIPD and DTD), the best modeling performance is achieved by porting models trained on large external datasets directly to the local setting. When deployed on tabular data, approaches such as random forest models seem to offer optimal prediction performance if proper pre-processing is deployed.

If a larger non-local dataset is readily available, our findings suggest an improvement in performance predicting such imbalanced outcomes using both non-local and local datasets for training a neural network with class weighting. In some cases, there could however be barriers against gaining access to a similar but larger dataset due to ethical or legal constraints.

Predicting outcomes where differences in clinical standard operating procedures, patient demographics or hospital resources creates a setting where outcomes are not directly comparable between hospital systems (e.g., hospital length of stay and long-term hospitalization), the best performing models evaluated by both ROC-AUC and F-score were trained only on local data, however marginally and with overlapping confidence intervals. While not suffering from high class imbalance this particular outcome most likely is heavily influenced by local workflows, policies and best practices. This again suggests that when assessing the optimal modeling approach, it is important to have domain knowledge of how incidence rates and workflows compare between hospital systems and datasets where models are trained. As such, scenarios where outcomes are not directly comparable, are best addressed through either de-novo local training or a transfer learning approach.

Care should be taken when comparing results directly between studies of this nature owing to the differences between methods, data, and selection of metrics for performance evaluation. Based on ROC-AUC, the primarily used metric amongst comparable studies, our best-performing mortality model (ROC-AUC 0.988) achieve excellent performance compared to other studies as evaluated by de Munter et al. covering 90 studies and 258 models (including TRISS) with ROC-AUC ranging between 0.59 and 0.98 (5) An advantage of a machine learning model over a conventional algorithm approaches such as TRISS worth noting, is the innate ability to still create a meaningful output despite of some missing information.

Comparing our results to the Norwegian survival prediction model in trauma (NORMIT) Jones et al. reported a ROC-AUC predicting mortality in Norwegian trauma population of 0.95 (6). Comparably, our best performing DTD-based de-novo mortality neural network model achieved 0.974 ROC-AUC.

A porting of NORMIT to a Finish trauma population showed a ROC-AUC of 0.83 (8) and ROC-AUC ranging 0.93–0.98 porting NORMIT 1 and 2 to a Swedish trauma population (35). Our TQIPID-based model achieved 0.971 ROC-AUC when porting, thus indicating similar performance. Our transfer learning approach achieved a higher ROC-AUC than the referred studies porting approaches and our own porting models with a ROC-AUC of 0.988. This indicates superiority in transfer learning compared to porting models directly. This finding is in adherence with a study on generalizability in multi-site COVID-19 screening, which demonstrated that site-specific customization comparatively improves performance (15).

We did not identify any directly comparable studies predicting hospitalization in days as a binary outcome (where length of stay longer than 2 commenced days) for entire trauma populations evaluated with ROC-AUC.

This study has several limitations. First, as is the case for any retrospective study, the underlying data quality is important and factors such as missing data could impact results. Secondly, the choice of model comparison methods is crucial. We chose to include threshold dependent metrics for evaluation with emphasis on the importance of sensitivity, or recall, when predicting mortality. This does, however, create another layer of complication in interpreting and comparing results with threshold moving by optimizing F-score from the DTD validation dataset. While viable in a process of comparing models, in an actual clinical application we would suggest probability calibration and sharing that probability with the clinician instead of a binary outcome. With calibrations, we still expect the models to rank similarly to the approach used for this study.

Third, optimally performing models could still fail in the clinical setting if there is a lack of explainable decision making by the model. Particularly neural networks have generally received criticism for “black boxing” how the model acts internally (36). While not being highest ranked in performance evaluation but still achieving good performance, Explainable Boosting Machine is worth highlighting for the conscious design based on interpretability (27).

In line with this, it should be noted that calculating feature importance is done by a variety of techniques which differ among models. As such, comparing feature importance between models should be done while inspecting each model with several explanation techniques (37). The variation in features and their ranking between models may thus be explained by the difference in explanation technique applied to the model rather than actual difference in the model decision making. Ultimately, such features as well as the model's performance should be benchmarked against seasoned clinical judgement.

Finally, results could be sensitive to hyper-parameters and training data, and it should furthermore be noted that changing the underlying data foundation (e.g., from tabular to unstructured data such as clinical chart note text) would likely result in alternative findings.

In conclusion, including data in the process of training machine learning models from the same domain but other healthcare systems, while implementing methods addressing class imbalance when relevant, likely improves performance when predicting infrequent clinical outcomes such as trauma mortality. When targeting balanced end-points not directly comparable between healthcare systems (e.g., hospital length of stay and long-term hospitalization), our results suggest that local de-novo training or transfer learning may be superior to porting models trained on external datasets. Collectively, the results thus suggest that optimal performance is critically dependent on domain knowledge and insight into the distributions of target variables in the dataset compared to external datasets.

## Data Availability

The original contributions presented in the study are included in the article/**Supplementary Materials**, further inquiries can be directed to the corresponding author. Project source code is available at the following GitHub repository https://github.com/a-millarch/trauma-ml-in-hospital.
