# Supplementary material for: Assessing optimal methods for transferring machine learning models to low-volume and imbalanced clinical datasets: experiences from predicting outcomes of Danish trauma patients
Source: Front Digit Health. 2023 Nov 2;5:1249258. doi: 10.3389/fdgth.2023.1249258 (PMC10656776; doi:10.3389/fdgth.2023.1249258)
Supplement: Supplementary file 1 [file Table1.docx]

| **Variable** | **Value range** | **Value description** |
| --- | --- | --- |
| Sex (female, male) | 0,1 | 0 female, 1 male |
| Hospital teaching status | University, community, non-teaching | Facility characteristic of the hospital describing teaching status |
| Hospital count of beds | > 600, 201-400, 401-600, <= 200 | Facility characteristic of the hospital describing facility size by counts of beds |
| Hospital ACS Trauma Center Verification level | I, II, III, IV | ACS level trauma center as described at: https://www.amtrauma.org/page/traumalevels |
| Comorbid condition: ADHD | 0,1 | Diagnosis present prior to injury (adherent to TQIP data definition) |
| Comorbid condition: Alcoholism | 0,1 | Diagnosis present prior to injury (adherent to TQIP data definition) |
| Comorbid condition: Angina pectoris | 0,1 | Diagnosis present prior to injury (adherent to TQIP data definition) |
| Comorbid condition: Anticoagulant treatment | 0,1 | Diagnosis present prior to injury (adherent to TQIP data definition) |
| Comorbid condition: Bleeding disorders | 0,1 | Diagnosis present prior to injury (adherent to TQIP data definition) |
| Comorbid condition: Chemotherapy | 0,1 | Diagnosis present prior to injury (adherent to TQIP data definition) |
| Comorbid condition: Cirrhosis | 0,1 | Diagnosis present prior to injury (adherent to TQIP data definition) |
| Comorbid condition: Chronic obstructive pulmonary disease | 0,1 | Diagnosis present prior to injury (adherent to TQIP data definition) |
| Comorbid condition: Cerebrovascular accident | 0,1 | Diagnosis present prior to injury (adherent to TQIP data definition) |
| Comorbid condition: Dementia | 0,1 | Diagnosis present prior to injury (adherent to TQIP data definition) |
| Comorbid condition: Diabetes (type 1 and 2) | 0,1 | Diagnosis present prior to injury (adherent to TQIP data definition) |
| Comorbid condition: Congestive heart failure | 0,1 | Diagnosis present prior to injury (adherent to TQIP data definition) |
| Comorbid condition: Hypertension | 0,1 | Diagnosis present prior to injury (adherent to TQIP data definition) |
| Comorbid condition: Myocardial infarction | 0,1 | Diagnosis present prior to injury (adherent to TQIP data definition) |
| Comorbid condition: Peripheral arterial disease | 0,1 | Diagnosis present prior to injury (adherent to TQIP data definition) |
| Comorbid condition: Mental disorders (schizophrenia, bipolar disorder, major depressive disorder, social anxiety, PTSD, antisocial personality disorder) | 0,1 | Diagnosis present prior to injury (adherent to TQIP data definition) |
| Comorbid condition: Chronic renal failure | 0,1 | Diagnosis present prior to injury (adherent to TQIP data definition) |
| Comorbid condition: Smoking tobacco every day or some days within past 12 months | 0,1 | Diagnosis present prior to injury (adherent to TQIP data definition) |
| Comorbid condition: Regular administration of oral or parental corticosteroid medications | 0,1 | Treatment present prior to injury (adherent to TQIP data definition) |
| Comorbid condition: Documented substance abuse (cannabis, hallucinogens, inhalents, opiods, sedatives, other) | 0,1 | Diagnosis present prior to injury (adherent to TQIP data definition) |
| Primary cause of injury | accident, assault, self-harm or other |  |
| Abbreviated injury scale (AIS) | AIS-codes | AIS-codes written as 12(34)(56).7, describing type, location and severity. Multiple AIS-codes might be present. |
| Pentrative injury | 0,1 | Derived from AIS position 3,4 equal 60 for penetrating trauma. If 1 then penetrating trauma. |
| Age in years | 0,130 | Age in years at the time of injury |
| Weight in kg | 3,215 | Weight in kilograms at the time of injury |
| Height in cm | 40,215 | Height in centimeters at the time of injury |
| Pre-hospital systolic blood pressure | 20,220 | Systolic blood pressure measured pre-hospital |
| Pre-hospital Glasgow Coma Score (total) | 1,15 | Total Glasgow Coma Score measure pre-hospital |
| In-hospital Glasgow Coma Score (total) | 1,15 | Total Glasgow Coma Score measure in-hospital |
| In-hospital systolic blood pressure | 20,220 | Systolic blood pressure measured in-hospital |
| In-hospital pulserate (BPM) | 30,300 | Pulserate in beats per minute measured in-hospital |
| In-hospital temperature in celsius | 15,50 | Temperature measured in celsius in-hospital |
| In-hospital pulseoximetry | 20,100 | Pulseoximetry SP02 measured in-hospital |
| In-hospital respiratory rate (BPM) | 0,100 | Respiratory rate in breaths per minute measured in-hospital |
| Injury severity score derived from AIS | 0,75 | Injury Severity Score calculated from AIS-scores by trauma care center |
| Difference between pre- and in-hospital Glasgow Coma Score | 0,15 | The absolute difference between pre- and in-hospital measurement |
| Difference between pre- and in-hospital systolic blood pressure measurement | 0,220 | The absolute difference between pre- and in-hospital measurement |
| In-hospital mortality | 0,1 | Binary outcome |
| Total length of stay longer than 2 days from admission | 0,1 | Binary outcome |

**Supplementary table 1:** Overview of variables with value ranges and short value description.
