# Supplementary material for: Assessing optimal methods for transferring machine learning models to low-volume and imbalanced clinical datasets: experiences from predicting outcomes of Danish trauma patients
Source: Front Digit Health. 2023 Nov 2;5:1249258. doi: 10.3389/fdgth.2023.1249258 (PMC10656776; doi:10.3389/fdgth.2023.1249258)
Supplement: Supplementary file 2 [file Datasheet1.docx]

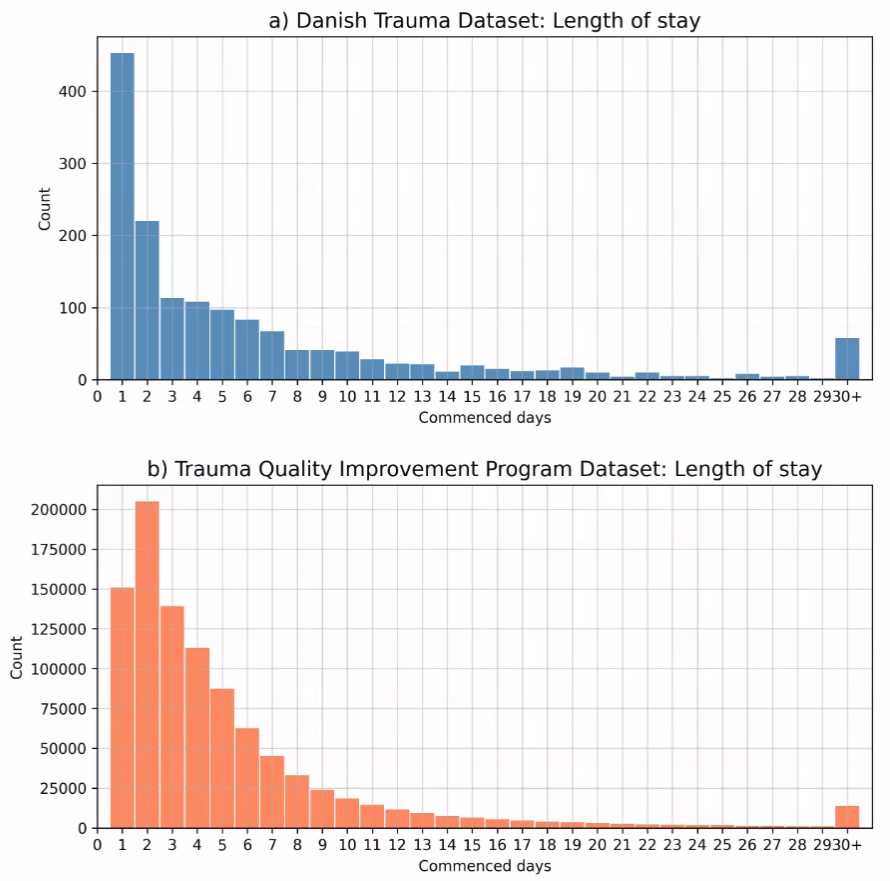


**Supplementary figure 1:** Distribution of length of stay in commenced days for a) the Danish Trauma Dataset (top figure) and for b) the Trauma Quality Improvement Program dataset (TQIPD, bottom figure)


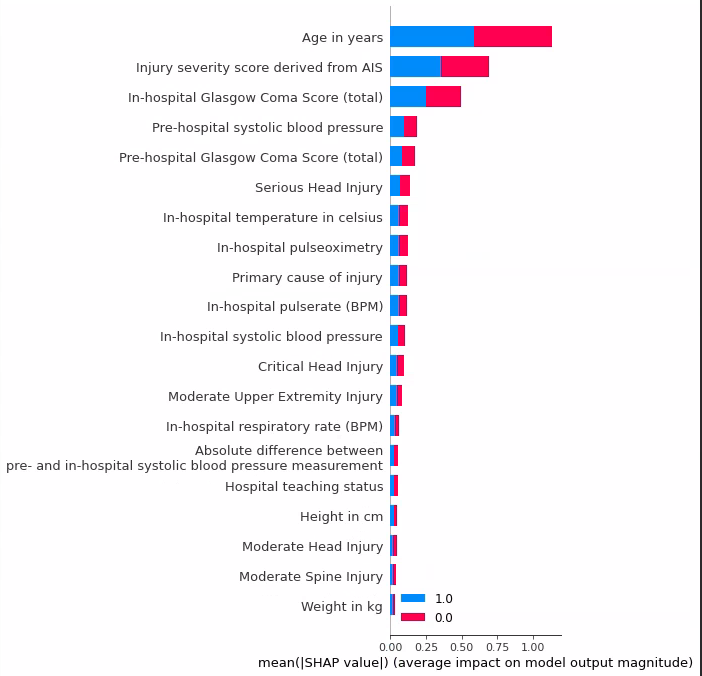


**Supplementary figure 2:** Shapley values for neural network mortality model trained on a mixed traning dataset consisting of a random forest selected subset of the Trauma Quality Improvement Program dataset and the Danish Trauma training dataset (DTD), retrained on DTD while adressing class imbalance by using a weighted loss function.


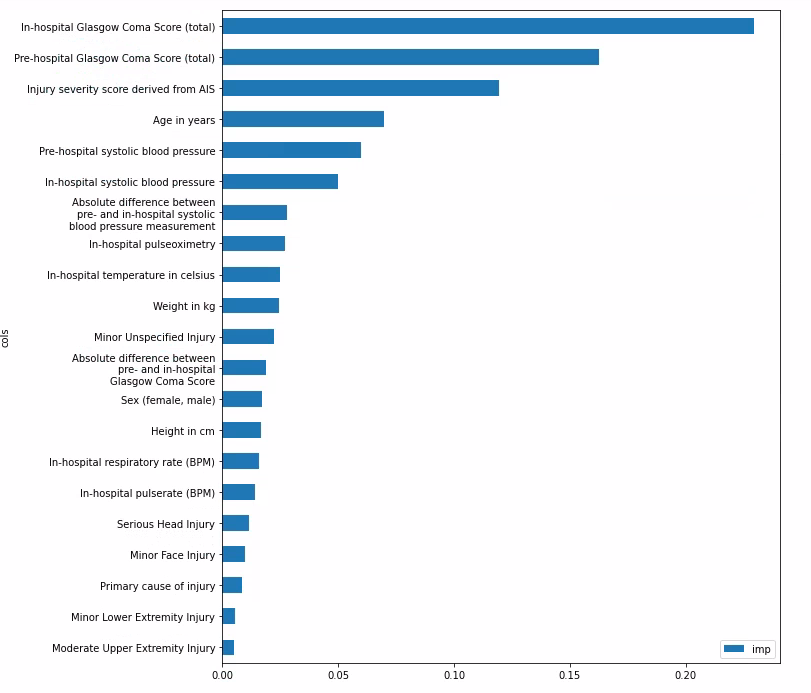
**Supplementary figure 3:** Feature importance for AdaBoost mortality model trained on Danish Trauma training dataset with applying Synthetic Minority-Oversampling Technique (SMOTE) and under-sampling by removing Tomek links


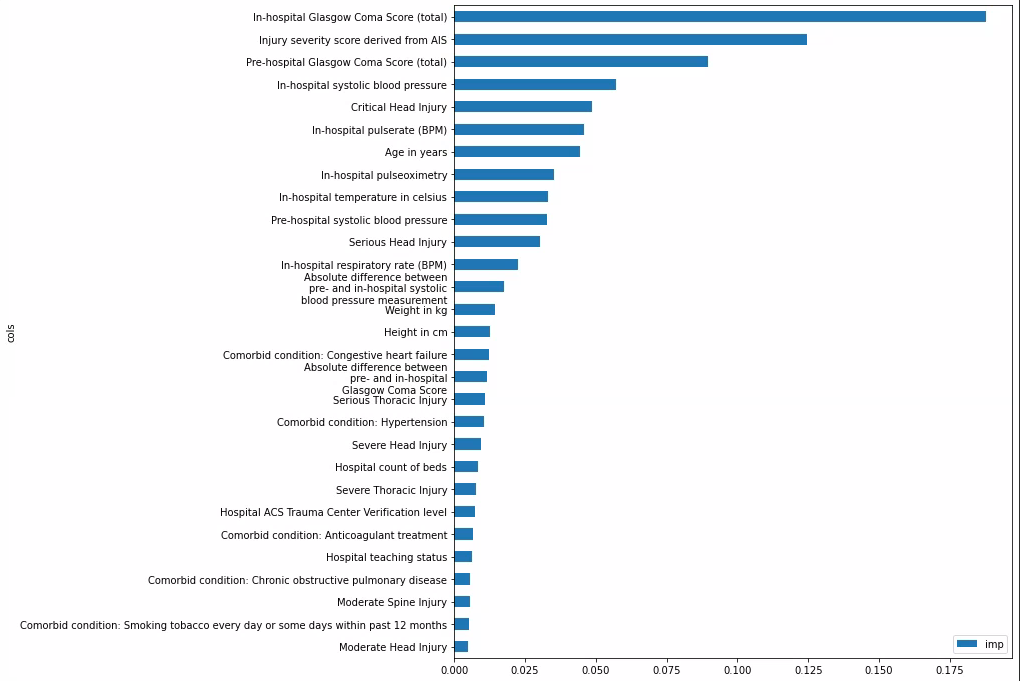


**Supplementary figure 4:**  Feature importance for random forest mortality model trained on the Trauma Quality Improvement Program dataset


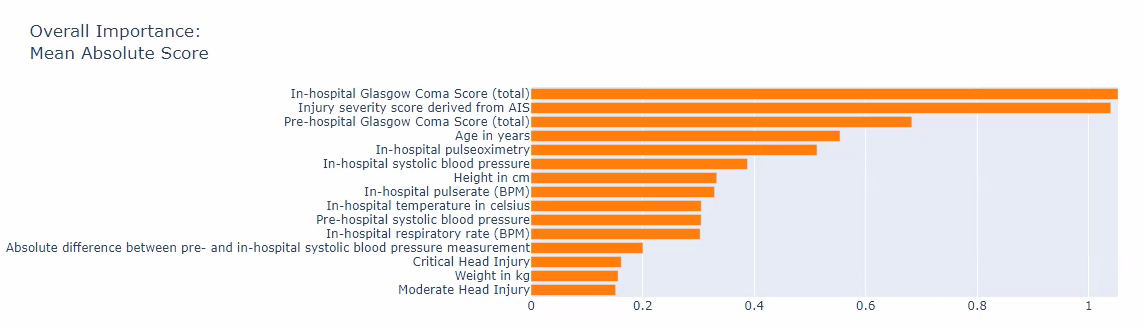


**Supplementary figure 5:** Summary of mean absolute score for Explainable Boosting Machine mortality model trained on a mixed traning dataset consisting of a random forest selected subset of the Trauma Quality Improvement Program dataset and the Danish Trauma training dataset while adressing class imbalance by using Synthetic Minority-Oversampling Technique (SMOTE)


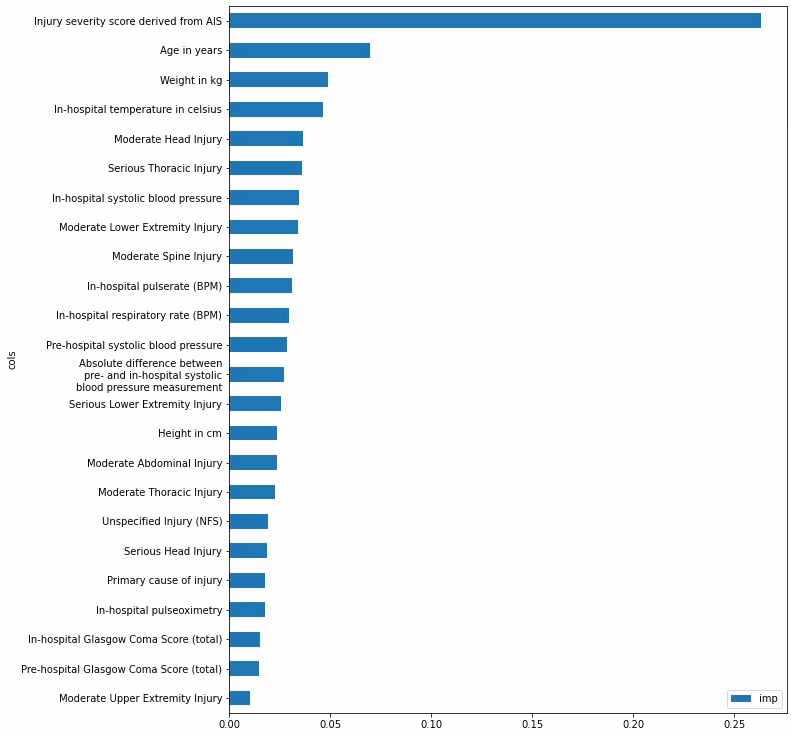


**Supplementary figure 6:** Feature importance for random forest long-term hospitalization model trained on Danish Trauma dataset.


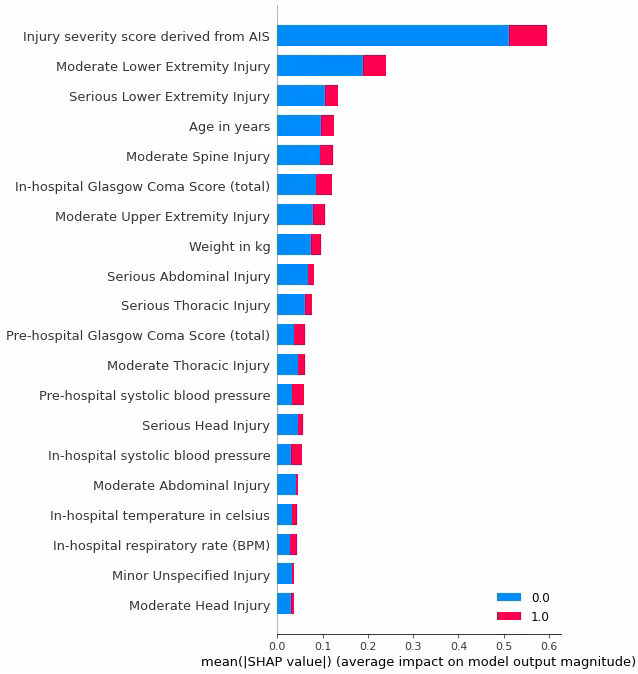


**Supplementary figure 7:** Shapley values for neural network long-term hospitalization model trained on a mixed traning dataset consisting of a random forest selected subset of the Trauma Quality Improvement Program dataset and the Danish Trauma training dataset (DTD) and then retrained on DTD.


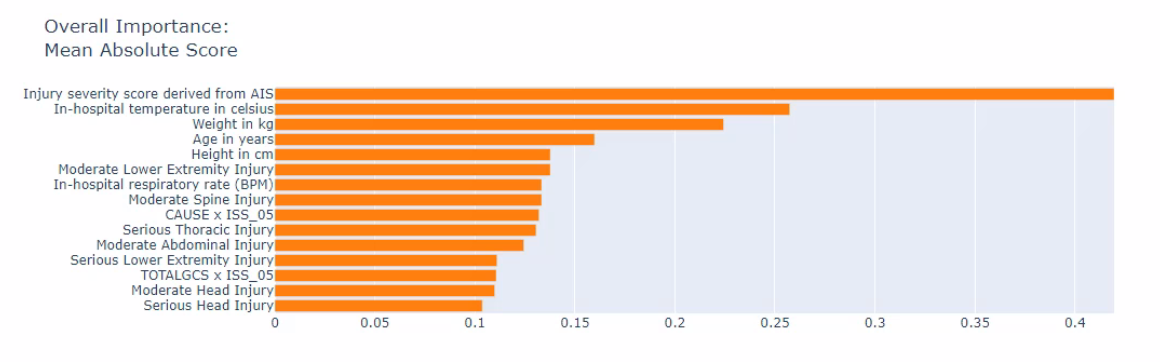


**Supplementary figure 8:** Summary of mean absolute score for Explainable Boosting Machine model trained on DTD.
